# Including the Eccentric Phase in Resistance Training to Counteract the Effects of Detraining in Women: A Randomized Controlled Trial

**DOI:** 10.1519/JSC.0000000000004039

**Published:** 2021-04-05

**Authors:** Giuseppe Coratella, Marco Beato, Luciano Bertinato, Chiara Milanese, Massimo Venturelli, Federico Schena

**Affiliations:** 1 Department of Biomedical Sciences for Health, University of Milan, Italy;; 2 School of Health and Sports Sciences, University of Suffolk, Ipswich, United Kingdom;; 3 Department of Neurological, Biomedicine and Movement Sciences, University of Verona, Verona, Italy; and; 4 CeRISM Research Center, University of Verona, Rovereto, Italy

**Keywords:** isokinetic, quadriceps, strength, hypertrophy, muscle architecture, vastus lateralis, fascicle length, DXA

## Abstract

Coratella, G, Beato, M, Bertinato, L, Milanese, C, Venturelli, M, and Schena, F. Including the eccentric phase in resistance training to counteract the effects of detraining in women: a randomized controlled trial. *J Strength Cond Res* 36(11): 3023–3031, 2022—The current study compared the effects of concentric-based (CONC), eccentric-based (ECC), and traditional concentric-eccentric (TRAD) resistance training on muscle strength, mass, and architecture and the postdetraining retention of the training-induced effects in women. Sixty women were randomly assigned to unilateral volume-equated CONC, ECC, or TRAD knee extension training or control (*N* = 15 per group). Before training, after an 8-week intervention period, and after an 8-week detraining period, isokinetic concentric, eccentric, and isometric torque were measured. In addition, thigh lean mass was assessed by dual X-ray absorptiometry and vastus lateralis thickness, pennation angle, and fascicle length by ultrasound. After training, concentric and isometric torque increased (*p* < 0.05) similarly in all groups, whereas eccentric torque increased more in ECC than that in CONC (+13.1%, effect size (ES): 0.71 [0.04–1.38]) and TRAD (+12.6%, ES: 0.60 [0.12–1.08]). Thigh lean mass increased in ECC (+6.1%, ES: 0.47 [0.27–0.67]) and TRAD (+3.1%, ES: 0.33 [0.01–0.65]). Vastus lateralis thickness and pennation angle increased (*p* < 0.05) similarly in all groups, whereas fascicle elongation was visible in ECC (+9.7%, ES: 0.92 [0.14–1.65]) and TRAD (+7.1%, ES: 0.64 [0.03–1.25]). After detraining, all groups retained (*p* < 0.05) similar concentric torque. ECC and TRAD preserved eccentric torque (*p* < 0.05), but ECC more than TRAD (+17.9%, ES: 0.61 [0.21–1.21]). All groups preserved isometric torque (*p* < 0.05), but ECC more than CONC (+14.2%, ES: 0.71 [0.04–1.38]) and TRAD (+13.8%, ES: 0.65 [0.10–1.20]). Thigh lean mass and vastus lateralis fascicle length were retained only in ECC (*p* < 0.05), pennation angle was preserved in all groups (*p* < 0.05), and thickness was retained in CONC and ECC (*p* < 0.05). Including the eccentric phase in resistance training is essential to preserve adaptations after detraining.

## Introduction

Resistance training is widely used to increase muscle strength and promote hypertrophy (28,41). Traditional resistance training protocols include the execution of both the concentric and eccentric phases and were reported as an effective way to induce gains in muscle strength (28) and hypertrophy (41). A different approach is basing the resistance training on performing either the concentric or the eccentric phase alone. Because the intensity usable during both traditional and concentric-based training depends on the maximal concentric strength, in practice an eccentric-based training allows supramaximal loads to be used, thus increasing the long-term mechanical stimuli (17,20). Notwithstanding, when performed in a volume-equated program (i.e., the combination of the load, load displacement, number of repetitions, and time under tension) (14), similar strength gains were reported after an eccentric-based, concentric-based, and traditional eccentric-concentric training, although the eccentric-based training was the only one that promoted a hypertrophic response (20). However, strength was assessed only as 1 repetition maximum (1RM), whereas it was observed that concentric-based or eccentric-based training led to greater specific increases in the concentric and eccentric strength, respectively (5). As such, including a more comprehensive strength evaluation by assessing concentric, eccentric, and isometric strength may provide further information about the capacity of each protocol to stimulate different strength exertion modalities (40). In addition, because the hypertrophic response was assessed indirectly as muscle girth (20), more accurate techniques and devices (e.g., lean mass assessed by dual X-ray absorptiometry [DXA] or muscle thickness by ultrasound) could be used to deepen this aspect (17).

The previous study that has examined the differences in muscle adaptations after concentric-based, eccentric-based, or traditional concentric-eccentric (TRAD) resistance training protocols and detraining did not examine muscle architecture (20). Muscle architecture is the geometrical arrangement of the muscle fascicles and includes both pennation angle and fascicle length (6). Greater pennation angle is due to greater amount of in-parallel sarcomeres and allows greater force to be exerted (6), whereas longer fascicles are due to more serial sarcomeres and favor faster contraction speed (13,16,19). Muscle architecture can be remodeled differently by an eccentric-based or concentric-based training protocol, with the former typically favoring fascicle elongation and the latter an increase in pennation angle (26). To further entangle the picture, the typical eccentric-based training response might be sex dependent because an increase in pennation angle was reported in women compared with men after an isokinetic eccentric-only training (15). Noteworthy, no study has recruited women to compare the effects of resistance training protocols on muscle architecture. In this regard, muscle architecture is sensitive to the mechanical stimulus induced by training, so unilateral rather than bilateral experimental model could better estimate the training-induced changes. Indeed, when resistance training is performed bilaterally, understanding how the load is distributed between the limbs is very difficult to be determined.

When training cessation (i.e., detraining) occurs, muscles can incur into a partial or total loss of the strength, hypertrophy, or muscle architecture owing to resistance training-induced adaptations (7). To possibly counteract the negative effects of detraining, it was shown that high-load training effectively retains the training-induced muscle strength gains (39). Remarkably, an eccentric-based training seemed more effective in retaining muscle strength, girth, and strength-endurance adaptations (20). A further study interestingly reported that an eccentric-based training retained both eccentric and concentric strength, whereas a concentric-based training only retained the concentric strength gains (44), highlighting the importance of assessing strength in different rather than single testing modalities.

Therefore, this study investigated the effects of a concentric-based, eccentric-based, and TRAD unilateral resistance training on total strength and isokinetic concentric, eccentric, and isometric peak torque, lean mass, muscle architecture, and the postdetraining retentions in women. Based on the above, it was hypothesized that the eccentric stimulus would be more effective in inducing and retaining the posttraining changes.

## Methods

### Experimental Approach to the Problem

This investigation was conceived as a parallel, 4 groups, pre-post, randomized controlled trial. Using a four-group restricted blocked randomization (computer-generated sequence), the subjects were randomized into 4 groups: concentric-only (CONC), eccentric-only (ECC), TRAD training, and control group (CTRL). One of the researchers without any contact or knowledge of the subjects completed the allocation and randomization of groups. The sample size was calculated a priori using statistical software (G-Power 3.1, Dusseldorf, Germany). Considering the study design (4 groups, 3 repeated measures), a medium effect size (ES) f = 0.25, a correlation among repeated measures *r* = 0.5, a nonsphericity correction ∈ = 1, an α–error = 0.05, and a required power 1 − β = 0.80, the total sample size resulted in 40 subjects. To overcome any drop in statistical power due to possible dropouts, we recruited 60 subjects, resulting in a posteriori statistical power 1 − β = 0.95.

To evaluate the knee extensors strength, isokinetic concentric, eccentric, and isometric peak torque were assessed. To evaluate hypertrophy, DXA scans of the thigh and vastus lateralis muscle thickness assessed by ultrasound were used. To evaluate muscle architecture, pennation angle and fascicle length were assessed on vastus lateralis.

**Figure 1.**
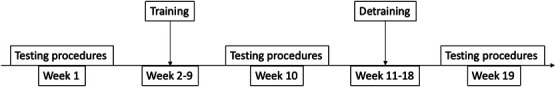
Study design.

This investigation lasted a total of 19 weeks, and the study procedures are shown in Figure 1. In week 1, the subjects were involved in 3 sessions. In the first session, they were familiarized with all intervention methods (CONC, ECC, and TRAD) and with the isokinetic testing modalities (concentric, eccentric, and isometric). In the second session, muscle architecture and DXA scans were assessed. In the third session, the isokinetic testing procedures were assessed. From week 2 to week 9, the subjects performed the intervention training. In week 10, posttraining testing procedures were assessed. Then, from week 11 to week 18, the subjects were involved in the detraining period and were instructed not to train. Finally, at week 19, the postdetraining testing procedures were assessed. Each testing assessment was performed by the same experienced operator.

### Subjects

Sixty healthy women were recruited among a university-based population (age: 22 ± 4 years, body mass: 60.2 ± 4.3 kg, and stature: 1.64 ± 0.06 m, body fat%: 22.9 ± 3.5; ± SD). The subjects were not engaged in a systematic resistance training for the previous 6 months but participated in one-to-three training sessions in different sports (e.g., volleyball, soccer, jogging, and tennis). For the entire duration of this study, the subjects were not allowed to participate in any other form of systematic resistance training. The subjects were instructed not to change their dietary habits, and a free software (www.myfitnesspal.com) was used to track the dietary intake every 5 weeks, with no significant difference observed between each assessment. In addition, the subjects did not use any oral contraceptive. Women with any hip, knee, or ankle disorder; muscle injury; and users of any drug were excluded from the study. All subjects signed written informed consent that was approved by the ethics committee of the University of Verona (N. 101-II-11) and were informed that they could withdraw from the study at any time. The procedures were conducted in accordance with the international ethical standards of the Declaration of Helsinki (1975 and further updates) for studies involving human subjects and approved by the University of Verona IRB.

### Procedures

An isokinetic dynamometer (Cybex Norm, Lumex, Ronkonkoma) was used to measure the knee extensors strength. The procedures followed previous protocols (17,18). In brief, the device was calibrated according to the manufacturer's recommendations, and the center of rotation was aligned with the tested knee. The subjects were seated on the dynamometer's chair, with their trunks slightly reclined backward and a hip angle of 85°. Two seatbelts secured the trunk and one strap secured the tested limb while the untested limb was secured by an additional lever. The testing measurements were preceded by a standardized warm-up, consisting of 3 sets × 10 repetitions of weight-free squats (10). Knee extensor strength was measured in concentric (1.05 rad·s^−1^), eccentric (−1.05 rad·s^−1^), and isometric (60°) modality (17). Each testing modality consisted of 3 maximal trials and was separated by 2 minutes of passive recovery. Strong standardized encouragements were provided to the subjects to maximally perform each trial. For each modality, during the familiarization session, the subjects were familiarized until 2 consecutive maximal tests differed each other less than 5%, in line with previous procedures (12). The reliability for the isokinetic parameters was calculated between-session, i.e., the familiarization and the pretraining testing assessment.

Total body and regional composition were evaluated using DXA, a total body scanner (QDR Explorer W, Hologic, MA; fan-beam technology, software for Windows XP version 12.6.1), according to the manufacturer's procedures. The scanner was calibrated daily against the standard supplied by the manufacturer to avoid possible baseline drift. Whole-body scanning time was about 7 minutes. Data were analyzed using standard body region markers: upper and lower extremities, head, and trunk (pelvic triangle plus chest or abdomen). In addition, the DXA scans were reanalyzed using nonstandard body region markers to define thigh segment. The thigh region was delineated by an upper border formed by an oblique line passing through the femoral neck to the horizontal line passing through the knee (17). All scanning and analyses were performed by the same experienced operator to ensure consistency. The lean mass of the trained limb was reported in data analysis.

**Figure 2.**
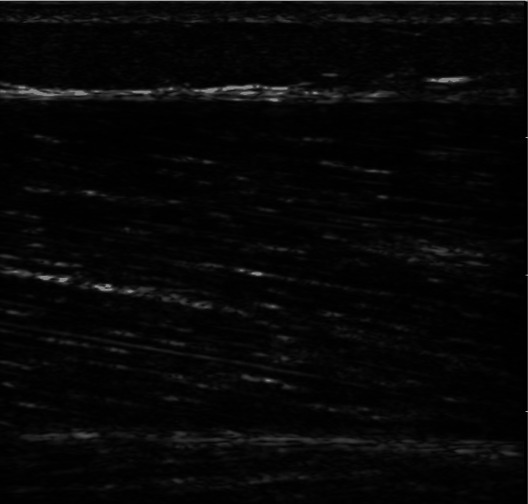
A typical ultrasound scan.

Muscle architecture was assessed in vivo at rest in vastus lateralis by B-mode ultrasound (LOGIQS7, GE, Fairfield, CT) with a 5-cm linear array probe (mod. 9 L, 3.1–10.0 MHz). The subjects lay supine on the examination bed with the hip joint extended and the knee joint almost fully extended (170° extension, with 180° full extension). The feet were immobilized by an operator to avoid any movement. The probe was held perpendicular to the skin surface by an expert operator, which ensured minimal pressure was applied to the muscle belly examined. No visually identifiable muscle compression was detected on the scan, as checked real time during the scan acquisition (16). A transmission gel was applied to improve acoustic coupling. Images were obtained along the vastus lateralis midsagittal plane, which included both superficial and deep aponeuroses, and the probe was oriented so that a number of clearly visible fascicles were captured. Careful manipulation was provided to align the transducer to the muscle fascicle plane and optimize the echogenicity of muscle fascicles (16). Two images were recorded at 50% of the thigh length, determined as the midpoint between the greater trochanter and the lateral condyle of the femur (4). The images were analyzed offline using an open-source computer program (ImageJ 1.44b, National Institutes of Health). Pennation angle was defined as the angle between the fascicle and aponeurosis. The measured angles were averaged and used for the analysis. Because at time of the data collection the extended field of view was not available, fascicle length was determined as the sum of the visible and an extrapolation of the nonvisible part (27). Finally, muscle thickness was measured as the distance between the superficial and deep aponeurosis (17). To minimize inconsistency in scan probe repositioning, identification of anatomical landmarks (fat, connective tissues, or blood vessels) that can be visualized in the same manner in every measure was performed because it is known that inhomogeneous changes in quadriceps muscle architecture may occur (24). A typical scan is shown in Figure 2. The subjects were instructed not to engage in any form of strenuous physical activity for the 24 hours before the muscle architecture assessment. *Excellent* between-day reliability usage was already shown (4), whereas here we calculated the between-scan, within-session reliability, taken from the pretraining session.

#### Intervention

The intervention lasted 8 weeks. In the first week, the subjects performed one training session because ECC would possibly have resulted in muscle damage (11), whereas from the second week on they performed 2 training sessions per week, for a total of 15 sessions. The unilateral dynamic constant external load knee extension training was performed on a gymnasium device (Leg extension Technogym, Cesena, Italy). To equalize the training volume, we manipulated the number of repetitions (sets × repetitions), the load considered as %1RM, fixing the consistent within-subject load angular displacement (approximately 85°) (17), and the time under tension (1.5 seconds) (17) for each phase (concentric or eccentric). Visual feedback (time = 1.5 seconds) was provided to the subjects to maintain the required time under tension (17,18). Therefore, for each training session, CONC performed 6 sets × 7 repetitions at 85%1RM; ECC performed 5 sets × 6 repetitions at 120%1RM; and TRAD performed 4 sets × 5 repetitions at 90%1RM, whereas CTRL did not train (20). Knee extensors 1RM was performed on the same device used for the training (Leg extension Technogym, Cesena, Italy), in line with previous procedures (17). During each repetition performed in CONC, an operator lowered the lever to relieve each subject from the eccentric phase; during each repetition performed in ECC, an operator lifted the lever to relieve each subject from the concentric phase; each repetition in TRAD was performed autonomously by the subjects without the help of any operator (20). The intervention was performed on the dominant limb, defined as the preferred limb to kick a ball (9). Each set was separated by 3 minutes of passive recovery. Each session was separated by at least 3 days. After the posttraining testing session, the subjects did not train for 8 weeks.

### Statistical Analyses

The statistical analysis was performed using a statistical software (SPSS 26.0, IBM, Armonk, NY). The normality of data was checked using the Kolmogorov-Smirnov test, and all data were found to be normal. The test-retest reliability was measured using an intraclass correlation coefficient (ICC) and interpreted as follows: α ≥ 0.9 = excellent; 0.9 > α ≥ 0.8 = good; 0.8 > α ≥ 0.7 = acceptable; 0.7 > α ≥ 0.6 = questionable; and 0.6 > α ≥ 0.5 = poor. To check the within-group and between-group differences in isokinetic concentric, eccentric, and isometric peak torque, quadriceps lean mass and vastus lateralis thickness, pennation angle, and fascicle length, mixed-factor analysis of variance was separately performed for each dependent parameter. In addition, to calculate between-group (4 groups: CONC, ECC, TRAD, and CTRL) differences in temporal adaptations (3 times: pretraining, posttraining, and postdetraining), data were log-transformed and analyzed using an analysis of covariance, considering prevalues as covariate. Multiple comparisons were calculated using the Bonferroni's correction. Significance was set at α < 0.05. Data are reported as mean with *SD*. Changes are reported as %change with 95% of confidence intervals (95% CIs) and Cohen's *d* effect size (ES) with 95% CI. Effect size was interpreted as follows: 0.00 to 0.19: *trivial*; 0.20 to 0.59: *small*; 0.60 to 1.19: *moderate*; 1.20 to 1.99: *large*; and ≥2.00: *very large*.

## Results

At baseline, no between-group difference was observed. The overall rate of compliance to the training program was 96.1% for CONC, 92.3% for ECC, and 93.7% for TRAD. *Excellent* reliability was found for concentric (ICC = 0.938), eccentric (ICC = 0.903), and isometric (ICC = 0.913) peak torque, vastus lateralis thickness (ICC = 0.917), and pennation angle (ICC = 0.925), and *good* reliability was found for fascicle length (ICC = 0.873).

**Figure 3.**
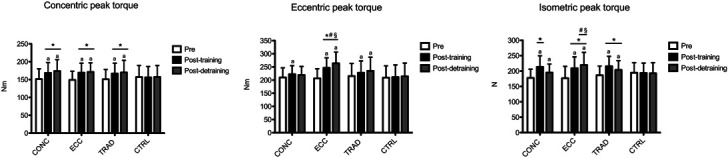
Time course of concentric, eccentric, and isometric peak torque is shown for each group. a: *p* < 0.05 vs. pre. *p < 0.05 vs. CTRL. #*p* < 0.05 vs. CONC. §*p* < 0.05 vs. TRAD. CONC = concentric-only training; ECC = eccentric-only training; TRAD = traditional concentric-eccentric training; CTRL = control group.

The results for concentric peak torque are shown in Figure 3. A time × group interaction was found (*p* < 0.001). Compared with pretraining, within-group analysis showed that concentric peak torque increased at posttraining in CONC (+11.2%, +7.2 to +14.5; ES: 0.61, 0.16–1.06), ECC (+13.4%, +9.4 to +17.4; ES: 0.80, 0.04–1.52), and TRAD (+10.6%, +6.6 to +13.9; ES: 0.62, 0.17–1.07), whereas CTRL did not show any change (*p* > 0.05). Between-group analysis showed that concentric peak torque increased similarly in all intervention groups, and such increases were greater than CTRL (*p* < 0.05). At postdetraining, concentric peak torque was still greater than that of pretraining in CONC (+14.6%, +9.9 to +19.2; ES: 0.75, 0.01–1.49), ECC (+14.7%, +9.4 to +19.4; ES: 0.90, 0.12–1.62), and TRAD (+12.6%, +7.9 to +17.2; ES: 0.62, 0.13–1.11). Between-group analysis showed that the concentric peak torque retention was similar in all intervention groups (*p* > 0.05) and greater than CTRL (*p* < 0.05).

The results for eccentric peak torque are shown in Figure 3. A time × group interaction was found (*p* < 0.001). Compared with pretraining, within-group analysis showed that eccentric peak torque increased at posttraining in CONC (+5.7%, +0.1 to +11.9; ES: 0.50, 0.02–0.98), ECC (+19.4%, +13.1 to +25.2; ES: 1.07, 0.28–1.80), and TRAD (+5.6%, +0.2 to +11.1; ES: 0.48, 0.01–0.95), whereas CTRL did not show any change (*p* > 0.05). Between-group analysis showed that eccentric peak torque increased more in ECC than CONC (+13.1, +3.9 to +21.8; ES: 0.71, 0.04–1.38) and TRAD (+12.6%, +3.7 to +21.8; ES: 0.60, 0.12–1.08), whereas CONC and TRAD did not differ from CTRL (*p* > 0.05). At postdetraining, eccentric peak torque was still greater compared with pretraining in ECC (+27.6%, +21.8 to +33.4; ES: 1.44, 0.60–2.20) and TRAD (+9.3%, +3.9 to +14.5; ES: 0.53, 0.15–0.91), but not in CONC (*p* > 0.05). Between-group analysis showed that the eccentric peak torque retention was greater in ECC than that in TRAD (+17.9%, +9.2 to +26.7; ES: 0.61, 0.21–1.21), and TRAD did not differ from CTRL (*p* > 0.05).

The results for isometric peak torque are shown in Figure 3. A time × group interaction was found (*p* < 0.001). Compared with pretraining, within-group analysis showed that isometric peak torque increased at posttraining in CONC (+20.1%, +13.5 to +26.6; ES: 1.12, 0.32–1.85), ECC (+18.3%, +11.5 + 25.1; ES: 0.86, 0.09–1.59), and TRAD (+15.6%, +9.1 to +21.5; ES: 0.95, 0.17–1.68), whereas CTRL did not show any change (*p* > 0.05). Between-group analysis showed that isometric peak torque increased similarly in all intervention groups (*p* > 0.05), and such increases were greater than CTRL (*p* < 0.05). At postdetraining, isometric peak torque was still greater compared with pretraining in CONC (+10.5%, +5.2 to +15.8; ES: 0.61, 0.24–0.98), ECC (+24.1%, +17.7 to +30.5; ES: 1.06, 0.27–1.80), and TRAD (+9.1%, +3.3 to +15.1; ES: 0.60, 0.13–1.07). Between-group analysis showed that the isometric peak torque retention was greater in ECC than that in CONC (+14.2%, +4.5 to +24.2; ES: 0.71, 0.04–1.38) and TRAD (+13.8%, +4.1 to +23.8; ES: 0.65, 0.10–1.20). Both ECC and TRAD differ from CTRL (*p* < 0.05), but not CONC (*p* > 0.05).

**Figure 4.**
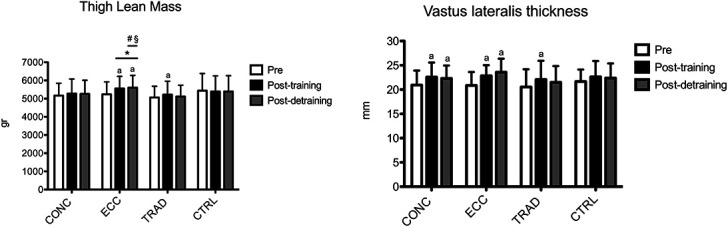
Time course of thigh lean mass and vastus lateralis thickness is shown for each group. a: *p* < 0.05 vs. pre. *p < 0.05 vs. CTRL. #*p* < 0.05 vs. CONC. §*p* < 0.05 vs. TRAD. CONC = concentric-only training; ECC = eccentric-only training; TRAD = traditional concentric-eccentric training; CTRL = control group.

The results for thigh lean mass are shown in Figure 4. A time × group interaction was found (*p* < 0.001). Compared with pretraining, within-group analysis showed that thigh lean mass increased at posttraining in ECC (+6.1%, +3.2 to +8.9; ES: 0.47, 0.27–0.67) and TRAD (+3.1%, +0.3 to +6.0; ES: 0.33, 0.01–0.65), but not in CONC and CTRL (*p* > 0.05). Between-group analysis showed that thigh lean mass increased similarly in ECC vs. TRAD (*p* > 0.05), but only ECC was different from CTRL (*p* < 0.05). At postdetraining, thigh lean mass was still greater compared with pretraining only in ECC (+7.0%, +4.5 to +9.6; ES: 0.54, 0.21 to 0.87), but not in TRAD (*p* > 0.05). Between-group analysis showed that the thigh lean mass retention was greater in ECC compared with all other groups (*p* < 0.05).

The results for vastus lateralis thickness are shown in Figure 4. No time × group interaction was found (*p* = 0.202), whereas a main effect for time (*p* < 0.001), but not for group (*p* = 0.760), was observed. Compared with pretraining, within-group analysis showed that vastus lateralis thickness increased at posttraining in CONC (+7.8%, +0.1 to +15.2; ES: 0.61, 0.01 to 1.21), ECC (+9.6%, +2.0 to +17.2; ES: 0.83, 0.06 to 1.55), and TRAD (+7.5%, 0.2–17.5; ES: 0.63, 0.04–1.22), whereas CTRL did not show any change (*p* > 0.05). At postdetraining, thickness was still greater than pretraining in CONC (+6.5%, +0.1 to +12.6%; ES: 0.58, 0.02 to 1.16) and ECC (+13.1%, +6.8 to +19.4; ES: 0.98, 0.20 to 1.71). Between-group analysis showed no difference at both posttraining and postdetraining.

**Figure 5.**
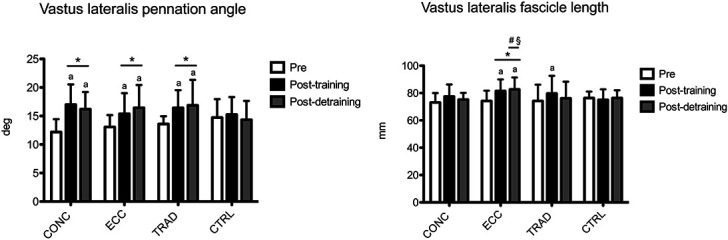
Time course of vastus lateralis pennation angle and fascicle length is shown for each group. a: *p* < 0.05 vs. pre. *p < 0.05 vs. CTRL. #*p* < 0.05 vs. CONC. §*p* < 0.05 vs. TRAD. CONC = concentric-only training; ECC = eccentric-only training; TRAD = traditional concentric-eccentric training; CTRL = control group.

The results for vastus lateralis pennation angle are shown in Figure 5. A time × group interaction was found (*p* = 0.019). Compared with pretraining, within-group analysis showed that pennation angle increased at posttraining in CONC (+39.4%, +20.8 to +57.9; ES: 1.62, 0.76–2.40), ECC (+17.6%, +0.2 to +35.6; ES: 0.79, 0.02–1.51), and TRAD (+20.8%, +4.2 to +37.5; ES: 1.20, 0.38–1.94), whereas CTRL did not show any change (*p* > 0.05). Between-group analysis showed that pennation angle increased similarly (*p* > 0.05) in all intervention groups, and such increases differ from CTRL (*p* < 0.05). At postdetraining, pennation angle was still greater compared with pretraining in CONC (+33.0%, +14.6 to +51.4; ES: 1.53, 0.68–2.29), ECC (+25.7%, +7.9 to +43.4; ES: 1.06, 0.27–1.79), and TRAD (+24.1%, +7.6 to +40.6; ES: 1.00, 0.21–1.73). Between-group analysis showed that the pennation angle retention was similar in all intervention groups (*p* > 0.05) and greater than CTRL (*p* < 0.05).

The results for vastus lateralis fascicle length are shown in Figure 5. A time × group interaction was found (*p* = 0.002). Compared with pretraining, within-group analysis showed that fascicle length increased at posttraining in ECC (+9.5%, +2.9 to +16.5; ES: 0.90, 0.12–1.61) and TRAD (+7.4%, +0.8 to +13.8; ES: 0.67, 0.05–1.27), whereas CONC and CTRL did not show any change (*p* > 0.05). Between-group analysis showed that fascicle length increased similarly (*p* > 0.05) in ECC and TRAD, but only ECC differed from CTRL (*p* < 0.05). At postdetraining, fascicle length was still greater compared with pretraining only in ECC (+10.7%, +5.8 to +16.6; ES: 1.01, 0.22–1.74). Between-group analysis showed that the fascicle length retention was greater in ECC compared with all other groups (*p* < 0.05).

## Discussion

This randomized controlled study investigated the effects of unilateral volume-equated CONC, ECC, and TRAD resistance training protocols on isokinetic knee extensors strength tested in different modalities, thigh lean mass and vastus lateralis thickness, and architecture adaptations and retentions in moderately active women. At posttraining, all groups increased similarly the concentric and isometric peak torque, whereas eccentric torque increased in ECC and TRAD more than CONC. At postdetraining, the increases in concentric torque were retained similarly in all groups. However, eccentric torque was still greater than baseline in ECC more than that in in TRAD, whereas CONC lost the training adaptations. Isometric torque was retained in all groups, but the retention was greater in ECC than that in TRAD and CONC. Thigh lean mass increased similarly in ECC and TRAD but not in CONC and was retained only in ECC. Vastus lateralis thickness increased similarly in all groups and was retained in CONC and ECC, but not in TRAD. Pennation angle increased and was retained similarly in all groups, whereas fascicle length increased in ECC and TRAD but was retained only in ECC. The present outcomes highlight the importance of the eccentric phase in resistance training to retain the muscle strength and structural adaptations in moderately active women.

It seems that the eccentric phase is fundamental to increase the eccentric strength (40), whereas similar gains in concentric and isometric torque were obtained by all groups. It is acknowledged that the current study cannot provide any mechanistic explanation. Interestingly, isokinetic eccentric-based training elicited longitudinal neuromuscular adaptations when maximum strength was tested in both eccentric and concentric modalities (44). By contrast, after a concentric-based training, such adaptations were found only in concentric but not eccentric testing modality (44). This may imply that the volitional drive elicited after the eccentric-based, but not concentric-based, training can be transferred to different strength modalities (44). In addition, eccentric-based training increases muscle excitability (46) and decreases the antagonist coactivation (38). Remarkably, an eccentric action may represent an unaccustomed task so that the muscle recruitment might be lower before starting a specific training and could increase more after an eccentric-based training (22). In this regard, eccentric actions are believed to increase the stimuli toward the type-II fibers that are less frequently stimulated, so that longitudinal eccentric-based training would preferentially increase type-II fibers' size, favoring eccentric strength gains (21). By contrast, a previous study reported greater gains in concentric strength and no difference in eccentric strength after an isokinetic concentric-only vs. eccentric-only knee extension training (5). Apart from the different training modality (i.e., isokinetic vs. dynamic constant external load), the authors did not measure the isometric peak torque and total strength, so this cannot be directly compared. Although traditional resistance training was shown to increase muscle strength in women (1), ECC was more effective than TRAD in increasing eccentric torque. It is possible that the relative eccentric stimulus was much higher in ECC (120% 1RM) than that in TRAD (90% 1RM), so that the eccentric strength may have benefited from the supramaximal intensity. It seems therefore that although resistance training increases concentric and isometric torque whatever the protocol, only the eccentric phase constitutes an effective stimulus to increase eccentric strength.

*Small* but significant increases in thigh lean mass were observed in ECC and TRAD, with no change in CONC, whereas vastus lateralis thickness similarly increased in all groups. To hypertrophic purposes, the inclusion of the eccentric phase was highly recommended by 2 different meta-analysis that showed a strong trend toward the superiority of eccentric-only vs. concentric-only training (40,42). Moreover, eccentric-based training protocols seem to effectively increase lean mass whatever the exercise modality, i.e., when performed using dynamic constant external load, isokinetic (17), or enhanced-eccentric modality using flywheel devices (9). This latter study also reported the effectiveness of TRAD squat to increase lower-limb lean mass (9). Similarly, traditional high-load training induced a rise in lean mass in women (1). Mechanistically, the repetitive mechanical sarcomere strain induced by the eccentric phase promotes muscle damage and a subsequent muscle reparation so that more proteins are added to avoid further damage (42). In addition, this phenomenon seems to specifically interest the type-II fibers (21), leading to greater increases in the overall muscle size. However, it is somehow surprising that no change in lean mass occurred in CONC, whereas increases in vastus lateralis thickness were observed. A previous study also showed no change in chest girth after CONC in resistance-trained men (20). By contrast, no difference in vastus lateralis volume was reported after concentric-based or eccentric-based leg press training (27). In addition, quadriceps volume equally increased after isokinetic concentric-only vs. eccentric-only training (5). It should be noted that inhomogeneous regional adaptations may occur in quadriceps (23,24), so that muscle thickness refers only to a specific quadriceps site, whereas thigh lean mass more comprehensively evaluate the whole thigh hypertrophy. Furthermore, because different testing modalities have been used to estimate muscle hypertrophy (e.g., DXA, ultrasound, magnetic resonance, and muscle girth), an actual comparison with the literature should take into account the different measurements sensitivity and the regional adaptations. However, these outcomes seem to confirm the small but significant superiority of the eccentric vs. concentric phase in promoting muscle hypertrophy (40,42).

Another interesting finding was that vastus lateralis pennation angle increased in all intervention groups similarly, although the within-group analysis retrieved *large* increases in CONC and TRAD and *moderate* in ECC. An increase in pennation angle should reflect a larger amount of in-parallel sarcomeres, which leads to larger physiological cross-sectional area (26). Such an adaptation is believed to be a typical concentric-based training-induced outcome (26), so the increases in CONC and TRAD were expected, while the increase in ECC might be surprising at a first glance. For example, no change in vastus lateralis pennation angle was observed after isokinetic or isoload eccentric-only knee extension training (4,17) or eccentric-only leg press training (27). However, the increases in pennation angle might have a sex influence. Although the aforementioned studies involved men only, an increase in vastus lateralis pennation angle after eccentric-only training was observed in women, but not in men (15). In addition, in a mixed women-men sample, isokinetic eccentric-only training led to increment in pennation angle (5). It is possible that the lower pennation angle baseline values in women vs. men might have increased the sensitivity to the resistance training, whatever the modality, given the negative correlation observed previously between the baseline values and the pennation angle increases (15). Therefore, the female population recruited here may explain the increase in pennation angle in ECC.

Similar *moderate* fascicle elongation was found in ECC and TRAD, with no change observed in CONC. Fascicle elongation was postulated to be accounted for an addition of in-series sarcomeres (26), although the serial sarcomerogenesis was recently questioned in favor of more comprehensive adaptations in the whole muscle-tendon unit (35). Whatever the mechanism, fascicle elongation seems to be a peculiarity of the eccentric-based training due to the repetitive strains fascicles undergo during each eccentric phase (17,26). Indeed, several studies reported simultaneous fascicle elongation after eccentric-based training and no change after concentric-based training (4,5,17,27). Conversely, an increase in fascicle length after both an isokinetic concentric-only or eccentric-only training was reported (5). However, the volume between the 2 intervention modalities was not matched and the knee extension movement started at 100°, so that fascicles were more strained at each repetition. Contrarily to pennation angle, no sex difference in vastus lateralis fascicle elongation was shown in women vs. men after an eccentric-only knee extension training (15). Longer fascicles increase the muscle contraction speed (6) and were associated with greater strength exerted at high-velocity torque (19), better performance in running sprint (33), and less time to achieve peak power (16). The fact that TRAD also showed fascicle elongation reinforces the rationale for including the eccentric phase when attempting increases in fascicle length.

The resistance training-induced adaptations include both neuromuscular and musculoskeletal changes (43), and a detraining period could possibly affect both. The posttraining effects may be transitory, and their persistence depends on the continuity of the training stimuli (36) and the duration of the detraining period, without any sex difference (7). The literature reports many cases where the resistance training-induced adaptations were retained. Primarily, this might depend on the load used during the training period. Indeed, high-load, but not low-load, traditional training maintained the strength increments after a 48-week detraining period (25). This was confirmed recently after a traditional training at ∼80% 1RM, followed by 20 weeks of inactivity (39). In addition, similar strength retention was observed after a maximal isokinetic concentric-only or eccentric-only training after 12 weeks of detraining (5). As such, the current high-load resistance training maintains the concentric and isometric strength gains, whatever the protocol. However, ECC and TRAD were able to retain the eccentric strength, although this was more visible in ECC. The capacity of an eccentric-based training to effectively stimulate the eccentric strength may play a key role because it seems that the eccentric vs. concentric strength retention could be per se greater (2). An eccentric-based training seems however superior in maintaining the strength gains. For example, only eccentric-based training was reported to retain the increase in bench press 1RM after 6 weeks of detraining (20). In addition, an eccentric-based intervention retained posttraining increases in concentric and eccentric strength, whereas a concentric-based training retained only the concentric strength, possibly because of a task-consistent retention in volitional drive (44). A further possible explanation for the superiority of ECC is the plasticity of the extracellular matrix, a bridge that transmits the force from the myofibers to the tendon (29). Indeed, after an eccentric-based exercise, extracellular matrix remodeling was shown after 27 but not 2 days (32), possibly explaining why ECC had greater eccentric strength retention after the detraining period. To summarize, these results indicate that high-load resistance training may retain the concentric and isometric strength gains after a detraining period. However, the eccentric phase must be included if the retention of the eccentric strength is warranted.

The current outcomes showed that only ECC retained the increase in thigh lean mass, whereas muscle thickness was retained in CONC and ECC. Similarly, an eccentric-based bench press training was the only to retain the gain in chest girth (20). To possibly explain the superiority of ECC in retaining the muscle size increases, different hypotheses are suggested. First, when undergoing repetitive eccentric contractions, some sarcomeres are disrupted (i.e., muscle damage) and this triggers a reparation process, the so-called repeated bout effect (31). This process possibly includes an increase in protein content (e.g., desmin) to confer the sarcomeres a protection against further mechanical stimuli (34). Because the repeated bout effect was shown to last up to 6 months during which no training was performed (37), it may be hypothesized that the protein addition may have a long-lasting effect. Second, an eccentric-based training showed a greater insulin-like growth factor and mechanogrowth factor and a simultaneous reduction in the myostatin gene expression (30). Such an anabolic profile is typically delayed up to several weeks after the end of a training period (8) and may also help to explain these results. Apart from the maintenance of specific regional adaptations, altogether, these mechanisms could account for the retention of the increase in thigh lean mass in ECC.

The time course of the architectural adaptations induced by different resistance training protocols has been poorly investigated. In this study, the increases in pennation angle observed in all groups were retained after the detraining period and the fascicle elongation was retained only in ECC. In a previous study, the increases in pennation angle after an eccentric-based and concentric-based training were retained after 12 weeks of detraining (5). Because greater pennation angle allows greater amount of contractile material to attach to the aponeurosis and contribute to the force generation (6), it is possible that the pennation angle retention may contribute to explain the total strength retention observed in all groups (5). This study also reported a retention in fascicle elongation after both protocols (5). Fascicle length seems to be reduced after an immobilization period (45), so a previous training protocol may be used to counteract such a decrease in length. Interestingly, it seems that pennation angle and fascicle length have different gene patterns (27), and this may imply different retention mechanisms.

This study comes with some limitations. First, the thigh lean mass was assessed by DXA, which cannot isolate the quadriceps muscle but takes into account the whole thigh segment. Second, muscle architecture was assessed only on vastus lateralis at midthigh. It is acknowledged that the within-muscle regional difference in muscle architecture may have resulted in different outcomes and also the examination of different quadriceps muscles (24). Third, all intervention groups included high-load protocols, and it is possible that the contraction-specific stimuli may also be load dependent. Fourth, the muscle activity was not measured, and it is acknowledged that it may have brought deeper information about the strength adaptations. Fifth, the dominant limb was used, and it is acknowledged that training dominant or nondominant limb could result in different findings (3). Finally, it is acknowledged that different populations may result in different outcomes.

In conclusion, this study shows that different volume-equated resistance training protocols performed by moderately active women increased concentric, eccentric, and isometric strength, albeit ECC led to superior gains in eccentric strength. Both ECC and TRAD increased thigh lean mass, underlining the importance of the eccentric phase in stimulating muscle hypertrophy, whereas the specific regional adaptations may have led to similar the increases in vastus lateralis thickness. The increases in vastus lateralis pennation angle were similar across all protocols, whereas fascicle length increased only in ECC and TRAD. After detraining, concentric and isometric strength were still retained in all groups, but greater retention in ECC was observed for eccentric strength. The increases in thigh muscle mass and fascicle length were retained only in ECC, whereas all groups retained the pennation angle increments.

**Practical Applications**

These findings have valuable impact in practice, especially when a detraining period could be planned in advance. The use of eccentric-based rather than traditional or concentric-based protocols permits the use of supramaximal loads, which may be determinant to retain both the muscle strength and structural training-induced adaptations. Indeed, although traditional resistance training protocol may provide a good overall maintenance of the strength adaptations, the inclusion of some sessions with an eccentric-based protocol may help to increase and better preserve the eccentric strength, useful for both sports' performance and daily life activities. In addition, longer fascicles turns into faster strength and power exertion (13,16), and its development and maintenance induced by eccentric-based protocols can be important when fast actions are required and need to be preserved. Finally, including the eccentric phase in resistance training seems to provide more beneficial hypertrophic effects and seems to be crucial not to lose the adaptations. To summarize, supramaximal loads seem critical to retain adaptations in eccentric strength and fascicle length that are lost with detraining after concentric and traditional training.
